# Supplementary material for: Generation of a Gluconobacter oxydans knockout collection for improved extraction of rare earth elements
Source: Nat Commun. 2021 Nov 18;12:6693. doi: 10.1038/s41467-021-27047-4 (PMC8602642; doi:10.1038/s41467-021-27047-4)
Supplement: Supplementary file 1 — Supplementary Information [file 41467_2021_27047_MOESM1_ESM.pdf]

Supplementary Information For:

Generation of a *Gluconobacter oxydans* knockout collection for improved extraction of rare earth elements

Alexa M. Schmitz[1], Brooke Pian[1], Sean Medin[1], Matthew C. Reid[2], Mingming Wu[1], Esteban Gazel[3], Buz Barstow[1†]

[1]Department of Biological and Environmental Engineering, Cornell University, Ithaca, NY 14853, USA

[2]School of Civil and Environmental Engineering, Cornell University, Ithaca, NY 14853, USA

[3]Department of Earth and Atmospheric Sciences, Cornell University, Ithaca, NY 14853, USA

[†]Corresponding author:

Buz Barstow, 228 Riley-Robb Hall, Cornell University, Ithaca, NY 14853; bmb35@cornell.edu

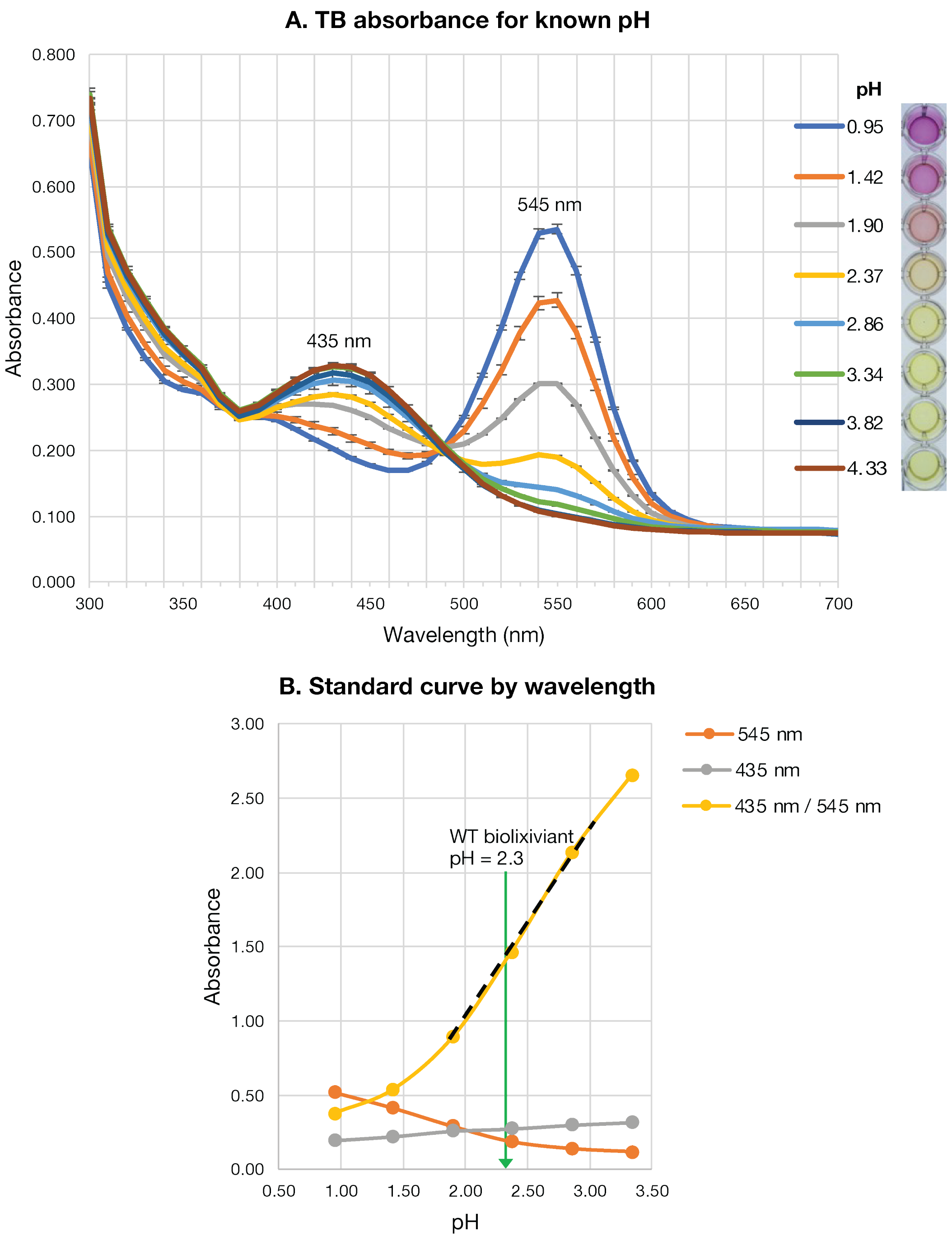

Supplementary Figure 1. Calibration data for Thymol Blue assay for media acidification. (**A**) Thymol Blue (TB) absorbance spectra for solutions of known pH. Error bars are standard deviation and n is 3 biological replicates. (**B**) Calibration curve to connect TB absorbance and pH. Black dashed line shows linearity of 435 nm / 545 nm between just below pH of 2.0 to a pH of 3.0. Green arrow indicates pH of the previously reported wild type *G. oxydans*-produced biolixiviant.

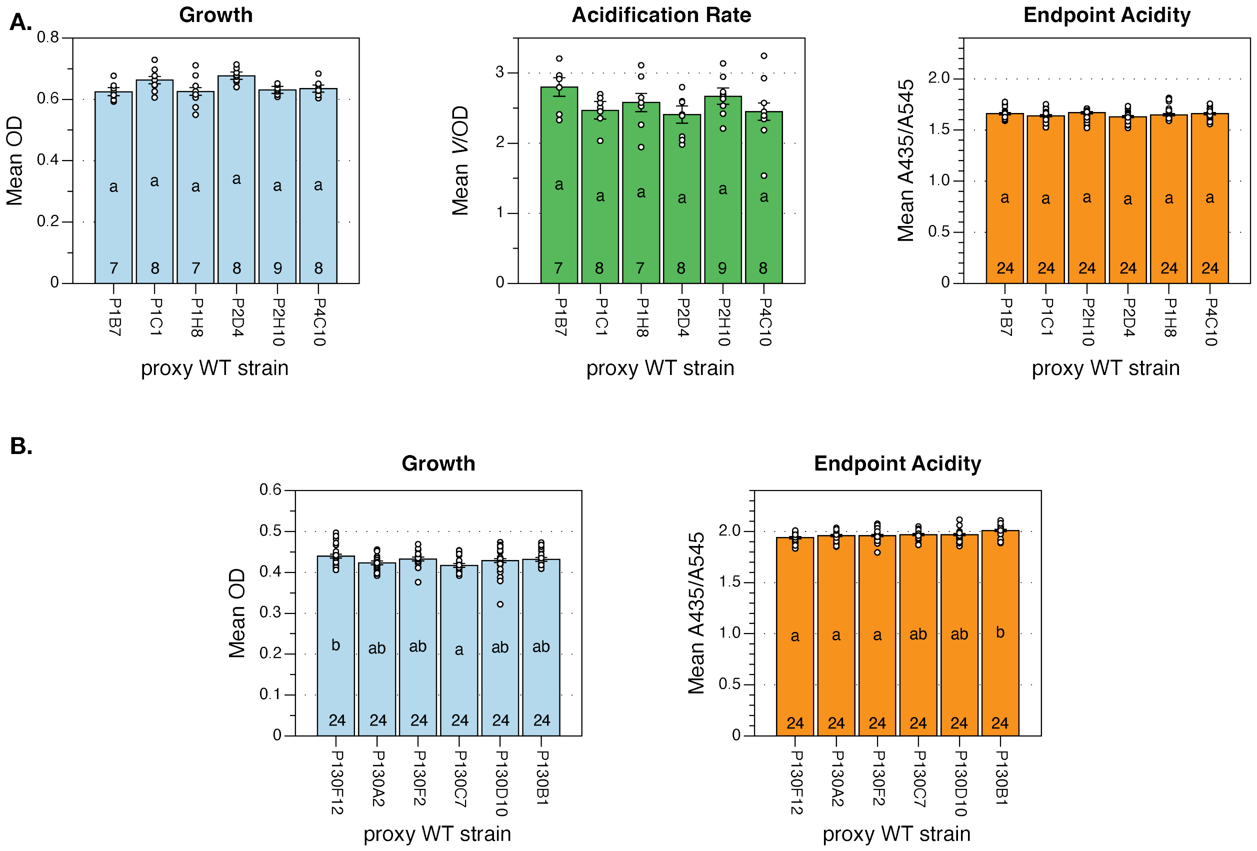

Supplementary Figure 2. Comparison of individual proxy WT disruption strains type A (**A**) and type B (**B**) growth (blue bars), initial acidification rate (green bars), and endpoint acidity (orange bars). Multiple comparisons were made for each set of proxy WT strains using the emmeans package in R with a Tukey *p*-value adjustment. Letters denote significance groups. Individual data points are shown as black circles. Error bars are standard error. The number of biological replicates for each strain measured for a particular phenotype is shown at the bottom of the corresponding bar.
