# Supplementary material for: Generation of a Gluconobacter oxydans knockout collection for improved extraction of rare earth elements
Source: Nat Commun. 2021 Nov 18;12:6693. doi: 10.1038/s41467-021-27047-4 (PMC8602642; doi:10.1038/s41467-021-27047-4)
Supplement: Supplementary file 3 — Description of Additional Supplementary Files [file 41467_2021_27047_MOESM3_ESM.pdf]

Description of Additional Supplementary Files

File Name: Supplementary Data 1
Description: *G. oxydans* B58 genome loci and feature names with transposon insertion sites.

File Name: Supplementary Data 2
Description: Gene Ontology Enrichment Data. (**A**) Non-disrupted genes in *G. oxydans* B58 mutant collection. (**B**) Disrupted genes with lower endpoint acidity. (**C**) Disrupted genes with higher endpoint acidity. (**D**) Disrupted genes with faster acidification. (**E**) Disrupted genes with slower acidification.

File Name: Supplementary Data 3
Description: Verification of *G. oxydans* B28 mutant identities by Sanger sequencing. (**A**) List of progenitor collection wells verified post-sequencing to confirm the results of location inference. (**B**) List of knockout collection wells verified post-sequencing of the condensed collection to verify strain confirmations. Wells with no "Illumina confirmed transposon coordinate" were unable to be confirmed by Illumina sequencing, but were hits in one of the screens, thus their transposon location was determined by Sanger sequencing.

File Name: Supplementary Data 4
Description: *G. oxydans* B58 condensed collection catalog.

File Name: Supplementary Data 5
Description: Results of initial acidification screens of *G. oxydans* B58 knockout collection. (**A**) All hits from Thymol Blue screen for end point pH and Bromophenol Blue (BPB) screen for acidification rate. H indicates that pH of biolixiviant was higher than the proxy wild-type, L indicates that the pH was lower. S indicates that rate of initial acidification was slower than proxy wild-type, F indicates that the rate was was faster. (**B**) Hits from Thymol Blue screen. (**C**) Hits from BPB screen. (**D**) Overlapping hits that appeared in both screens.

File Name: Supplementary Data 6
Description: Validation results for initial hits identified by acidification screens of *Gluconobacter oxydans* knockout collection. (**A**) All hits. H indicates that pH of biolixiviant was higher than the proxy wild-type, L indicates that the pH was lower. S indicates that rate of initial acidification was slower than proxy wild-type, F indicates that the rate was was faster. (**B**) All significant hits from end point and acidification rate screens. (**C**) Significant hits from TB end point pH screen. (**D**) Significant hits from BPB acidification rate screen. (**E**) Significant hits from TB screen represented in **Fig. 2D**. (**F**) Significant hits from BPB screen represented in **Fig. 2E**.
