# Supplementary material for: Generation of a Gluconobacter oxydans knockout collection for improved extraction of rare earth elements
Source: Nat Commun. 2021 Nov 18;12:6693. doi: 10.1038/s41467-021-27047-4 (PMC8602642; doi:10.1038/s41467-021-27047-4)
Supplement: Supplementary file 10 — Reporting Summary [file 41467_2021_27047_MOESM10_ESM.pdf]

nature portfolio

Corresponding author(s): Buz Barstow

Last updated by author(s): Aug 3, 2021

Reporting Summary

Nature Portfolio wishes to improve the reproducibility of the work that we publish. This form provides structure for consistency and transparency in reporting. For further information on Nature Portfolio policies, see our Editorial Policies and the Editorial Policy Checklist.

Statistics

For all statistical analyses, confirm that the following items are present in the figure legend, table legend, main text, or Methods section.

n/a	Confirmed	
☐	☒	The exact sample size (n) for each experimental group/condition, given as a discrete number and unit of measurement
☐	☒	A statement on whether measurements were taken from distinct samples or whether the same sample was measured repeatedly
☐	☒	The statistical test(s) used AND whether they are one- or two-sided<br>*Only common tests should be described solely by name; describe more complex techniques in the Methods section.*
☒	☐	A description of all covariates tested
☒	☐	A description of any assumptions or corrections, such as tests of normality and adjustment for multiple comparisons
☐	☒	A full description of the statistical parameters including central tendency (e.g. means) or other basic estimates (e.g. regression coefficient) AND variation (e.g. standard deviation) or associated estimates of uncertainty (e.g. confidence intervals)
☐	☒	For null hypothesis testing, the test statistic (e.g. F, t, r) with confidence intervals, effect sizes, degrees of freedom and P value noted<br>*Give P values as exact values whenever suitable.*
☒	☐	For Bayesian analysis, information on the choice of priors and Markov chain Monte Carlo settings
☒	☐	For hierarchical and complex designs, identification of the appropriate level for tests and full reporting of outcomes
☒	☐	Estimates of effect sizes (e.g. Cohen's d, Pearson's r), indicating how they were calculated

Our web collection on statistics for biologists contains articles on many of the points above.

Software and code

Policy information about availability of computer code

Data collection	Plate reader data was collected by Gen5 microplate reader and imager software, version 3.05. ICP-MS data was collected with MassHunter, version 4.5.
Data analysis	Genome sequencing data was analyzed and assembled with Trimmomatic, SPAdes, QUAST, BUSCO and RAST. Gene enrichment was determined by analyzing the full set of protein models with DIAMOND, InterProScan, and the BioConductor topGO package. Sequencing data for generating the knockout collection was analyzed using the kosudoku package. Plate reader data was analyzed by Gen5 microplate reader and imager software, and Microsoft Excel version 16.51. ICP-MS data was analyzed with MassHunter, version 4.5 and Microsoft Excel version 16.51. Pairwise comparisons were made using the emmeans package in R. All other data analysis was done in Microsoft Excel version 16.51.

For manuscripts utilizing custom algorithms or software that are central to the research but not yet described in published literature, software must be made available to editors and reviewers. We strongly encourage code deposition in a community repository (e.g. GitHub). See the Nature Portfolio guidelines for submitting code & software for further information.

Data

Policy information about availability of data

All manuscripts must include a data availability statement. This statement should provide the following information, where applicable:

- Accession codes, unique identifiers, or web links for publicly available datasets
- A description of any restrictions on data availability
- For clinical datasets or third party data, please ensure that the statement adheres to our policy

Experimental data generated in this study are provided in the Supplementary Information/Source Data file. Raw sequencing data are available through Corn

ell University eCommons: https://doi.org/10.7298/7s81-5t81. The assembled G. oxydans NRRL B58 genome has been deposited at DDBJ/ENA/GenBank under the accession JAIPVW000000000. The version described in this paper is version JAIPVW010000000: https://www.ncbi.nlm.nih.gov/nuccore/JAIPVW000000000.

Field-specific reporting

Please select the one below that is the best fit for your research. If you are not sure, read the appropriate sections before making your selection.

- [x] Life sciences
- [ ] Behavioural & social sciences
- [ ] Ecological, evolutionary & environmental sciences

For a reference copy of the document with all sections, see nature.com/documents/nr-reporting-summary-flat.pdf

Life sciences study design

All studies must disclose on these points even when the disclosure is negative.

Sample size	Trial experiments were performed for each experiment that served as a basis for choosing sample size. No formal statistical analysis was done for determining sample size.
Data exclusions	In some cases, as described in the text, data was excluded as a result of poor bacterial growth.
Replication	For the high-throughput screen, individual plates were run more than once to verify reproducibility. The size of the screen precluded an entire replication; however all plates were run successfully at least once. All other experiments were done at least twice with similar results.
Randomization	Samples were randomly distributed in 96-well microplates for all experiments.
Blinding	Investigators were blind to the contents of each plate during collection and analysis of the high-throughput screening data for the identification of preliminary gene targets. For all other experiments, samples were given identifiers based on randomized location in microplates and only those identifiers were known at time of data collection and analysis.

Reporting for specific materials, systems and methods

We require information from authors about some types of materials, experimental systems and methods used in many studies. Here, indicate whether each material, system or method listed is relevant to your study. If you are not sure if a list item applies to your research, read the appropriate section before selecting a response.

Materials & experimental systems

n/a	Involved in the study	
☒	☐	Antibodies
☒	☐	Eukaryotic cell lines
☒	☐	Palaeontology and archaeology
☒	☐	Animals and other organisms
☒	☐	Human research participants
☒	☐	Clinical data
☒	☐	Dual use research of concern

Methods

n/a	Involved in the study	
☒	☐	ChIP-seq
☒	☐	Flow cytometry
☒	☐	MRI-based neuroimaging
